# Supplementary material for: The Synthesis of Bio-Based Michael Donors from Tall Oil Fatty Acids for Polymer Development
Source: Polymers (Basel). 2022 Sep 30;14(19):4107. doi: 10.3390/polym14194107 (PMC9572469; doi:10.3390/polym14194107)
Supplement: Supplementary file 1 [file polymers-14-04107-s001.zip › polymers-1918628-SM.pdf]

Table S1. Possible structures that could correspond to the peaks in the MALDI-TOF spectrum (see Figure 4.)

Synthesized component	Peaks, m/z	Theoretically probable structures	Molar mass (X+Na+), g/mol
E[IR]TOFA_BD	425.33	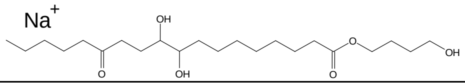	425.55
	481.31	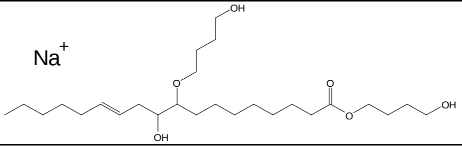	481.66
	483.41	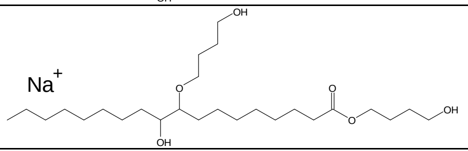	483.68
	497.39	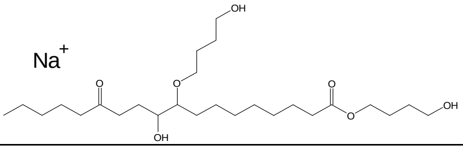	497.66
	795.65	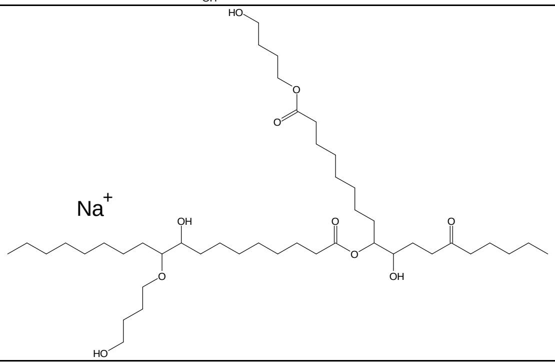	796.12
E[IR]TOFA_BD_AA	677.39	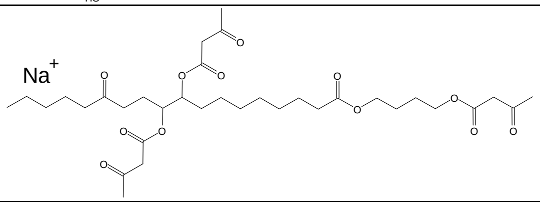	677.77
	733.45	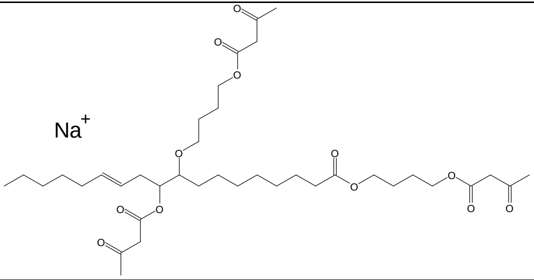	733.88

Synthesized component	Peaks, m/z	Theoretically probable structures	Molar mass (X+Na+), g/mol
	735.47	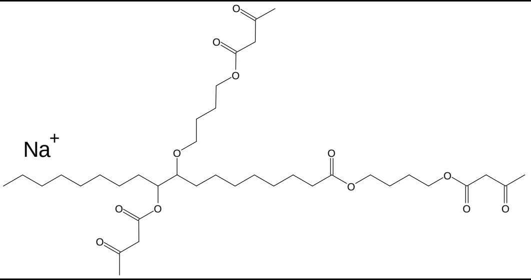	735.90
	749.45	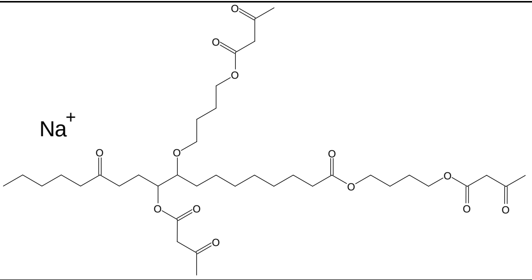	749.88
	1131.74	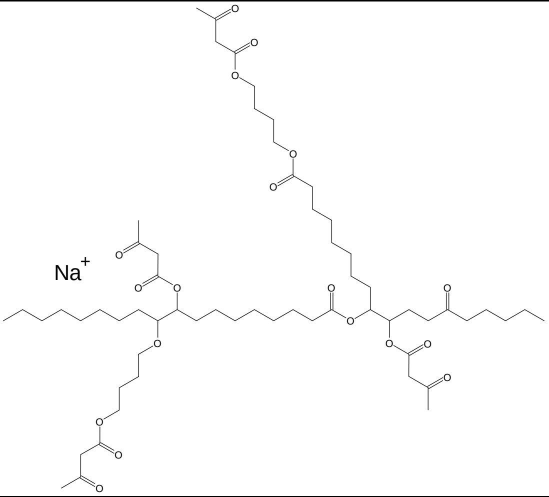	1132.41
E^{IR}TOFA_TMP	455.34	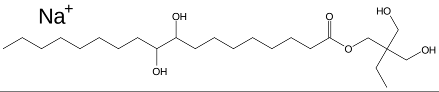	455.62
	469.33	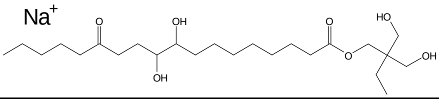	469.61
	569.42	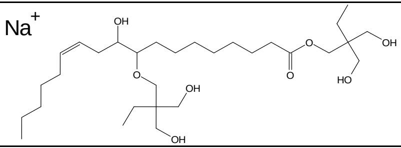	569.77
	571.43	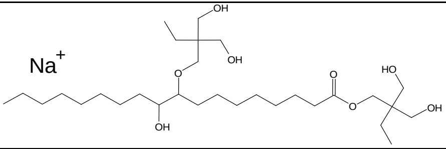	571.78
	585.41	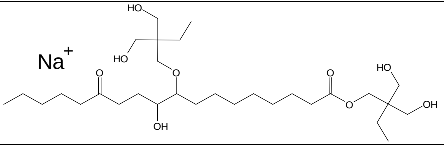	585.77

**Synthesized component**	**Peaks, m/z**	**Theoretically probable structures**	**Molar mass (X+Na+), g/mol**
	719.51	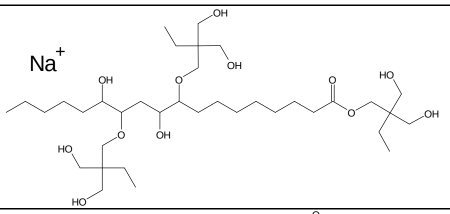	719.94
E^{IR}TOFA_TMP_AA	409.19	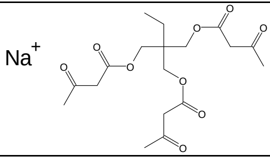	409.38
	804.44	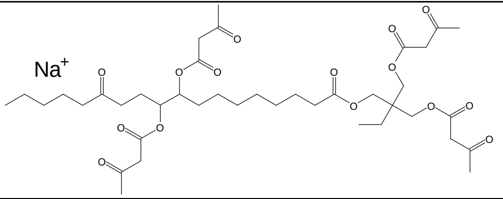	805.90
	989.56	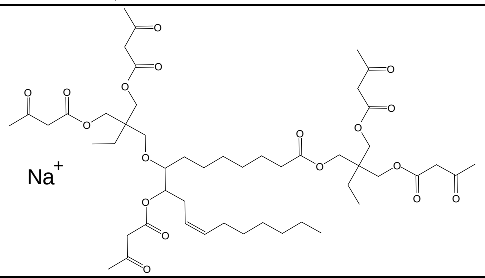	990.13
	991.57	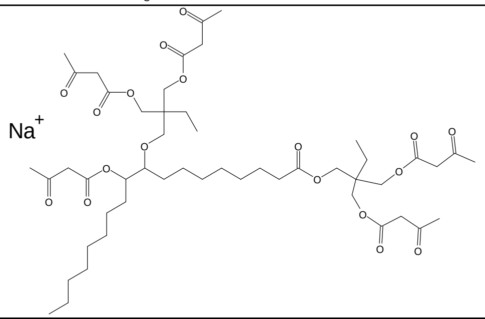	992.15
	1005.55	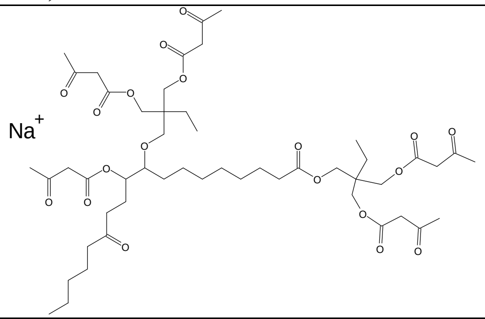	1006.13

Synthesized component	Peaks, m/z	Theoretically probable structures	Molar mass (X+Na+), g/mol
	1391.72	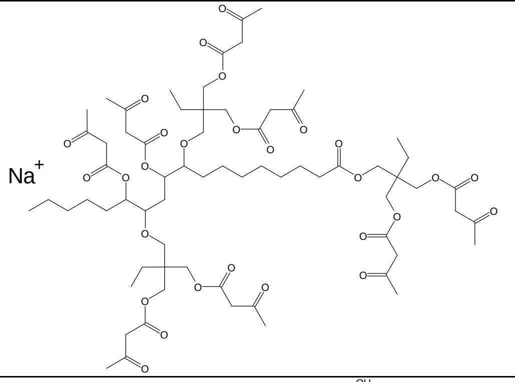	1392.53
L3300	463.31	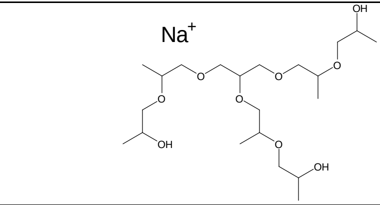	463.56
L3300_AA	715.40	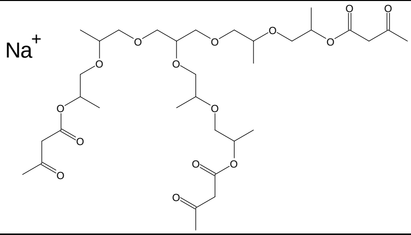	715.78
